# Plasma lncRNA expression profile as a prognostic tool in *BRAF-*mutant metastatic melanoma patients treated with BRAF inhibitor

**DOI:** 10.18632/oncotarget.26989

**Published:** 2019-06-11

**Authors:** Tomasz Kolenda, Piotr Rutkowski, Michał Michalak, Katarzyna Kozak, Kacper Guglas, Marcel Ryś, Łukasz Galus, Sebastian Woźniak, Iwona Ługowska, Aleksandra Gos, Anna Teresiak, Andrzej Mackiewicz, Katarzyna Lamperska, Jacek Mackiewicz

**Affiliations:** ^1^ Department of Cancer Immunology, Chair of Medical Biotechnology, Poznan University of Medical Sciences, Poznan, Poland; ^2^ Laboratory of Cancer Genetics, Greater Poland Cancer Centre, Poznan, Poland; ^3^ Postgraduate School of Molecular Medicine, Medical University of Warsaw, Warsaw, Poland; ^4^ Department of Soft Tissue/Bone Sarcoma and Melanoma, Maria Sklodowska-Curie Institute - Oncology Center, Warsaw, Poland; ^5^ Department of Computer Science and Statistics, University of Medical Sciences, Poznan, Poland; ^6^ Department of Medical and Experimental Oncology, Heliodor Swiecicki Clinical Hospital, Poznan University of Medical Sciences, Poznan, Poland; ^7^ Department of Chemotherapy, Greater Poland Cancer Centre, Poznan, Poland; ^8^ Early Phase Clinical Trials Unit, Maria Sklodowska-Curie Institute - Oncology Center, Warsaw, Poland; ^9^ Department of Translational Oncology, Maria Sklodowska-Curie Institute - Oncology Center, Warsaw, Poland; ^10^ Department of Diagnostics and Cancer Immunology, Greater Poland Cancer Centre, Poznan, Poland; ^11^ Department of Biology and Environmental Sciences, Poznan University of Medical Sciences, Poznan, Poland

**Keywords:** melanoma, BRAF mutation, BRAF inhibitor, lncRNA, liquid biopsy

## Abstract

Long non-coding RNAs (lncRNA) are dysregulated in many cancer types. Abnormal baseline levels of these lncRNAs display diagnostic and prognostic potential in cancer patients. The aim of this study was to evaluate the prognostic value of plasma lncRNAs in *BRAF*-mutant advanced melanoma patients treated with a BRAF inhibitor. Total RNA was isolated from plasma samples collected from 58 advanced BRAF-mutant melanoma patients and 15 healthy donors. The expression levels of 90 lncRNAs were estimated using the LncProfiler qPCR Array Kit (SBI) and LightCycler 96 (Roche). LncRNA expression levels correlated with responses to the BRAF inhibitor (vemurafenib) treatment. The patients were stratified into three groups based on their lncRNA levels with various lncRNA expressions (low, medium, and high). A Cox proportional hazards regression model was used to determine the lncRNAs that were significantly associated with both progression-free and overall survivals (PFS and OS, respectively) in patients receiving vemurafenib. The expression level of 12 lncRNAs was down-regulated, while five lncRNAs were up-regulated in melanoma patients compared to healthy donors. Kaplan-Meier analysis showed that upregulation or downregulation of 11 and 16 different lncRNAs were associated with longer median PFS and OS, respectively. Further analysis demonstrated that the baseline lncRNAs for IGF2AS, anti-Peg11, MEG3, Zeb2NAT are independent prognostic factors in *BRAF*-mutant advanced melanoma patients treated with vemurafenib. Evaluation of plasma lncRNAs expression level for advanced melanoma diagnosis and prognosis evaluation appears to be a safe and valuable method; however, this method requires further validation in larger cohorts and randomized trials.

## INTRODUCTION

The treatment landscape in melanoma is rapidly changing. The introduction of immunotherapy and targeted therapy to standard treatment regimens has improved the prognosis of melanoma patients. However, despite the significant progress in melanoma treatment modalities, a number of patients still do not respond to the therapy or develop resistance to the medicinal products and die early. Vemurafenib, the v-Raf murine sarcoma viral oncogene homolog B (BRAF) inhibitor was the first new generation drug approved for skin melanoma treatment. In a randomized phase 3 trial, it extended median overall survival (OS) for patients by four months compared to chemotherapy in BRAF-mutated advanced melanoma patients [1]. Subsequent clinical studies showed that addition of a mitogen-activated protein kinase kinase (MEK) inhibitor to the BRAF inhibitor translates into further survival improvement [2–7]. Various mechanisms of primary and secondary resistance to BRAF and MEK inhibitors have been described [8]. Moreover, novel prognostic biomarkers have been identified in patients treated with targeted therapy (BRAF and MEK inhibitors) and immunotherapy (anti-programmed cell death protein [PD]1 and anti-cytotoxic T-lymphocyte–associated antigen [CTLA]4) [9–11]. Accordingly, continuous research, including predictive biomarker identification, is required to guide physicians’ decision on whether targeted therapy or immunotherapy should be applied for the first-line treatment in BRAF mutant patients in order to improve their survival.

Liquid biopsies provide non-invasive and easy sources of circulating RNAs [12–14]. Different types of both shorter and lncRNAs are detectable in the whole blood, serum, and plasma [15]. The lncRNAs are molecules >200 nucleotides and are actively transcribed but do not encode proteins. LncRNA molecules possess many functional domains, such as RNA, DNA, or protein binding sites and may play crucial physiological roles in controlling transcription and post-transcriptional processes and protein translation and/or may influence epigenetic modifications. They participate in proliferation, apoptosis, stress responses, and regulation of cell metabolism or cell phenotype [16]. LncRNAs are candidates for a new class of biomarkers [16, 17]. However, little is known about the role of circulating lncRNAs.

Application of various types of RNA as biomarkers is not new, but there is still a lack of specified diagnostic panels. Assessment of lncRNA expression in various specimens, such as tissue, urine, peripheral blood, serum, saliva, and/or urine may be easily done using different diagnostics methods, but real-time quantitative reverse transcription polymerase chain reaction (qRT-PCR) is the most frequently used method [17].

Accordingly we have analyzed the expression of 90 lncRNAs that are potentially connected with cancer. LncRNAs were assessed in the plasma of pretreated BRAF-mutated unresectable stage III and IV cutaneous melanoma patients and healthy donors in order to assess their potential as biomarkers of BRAF inhibitor treatment efficacy. We show that melanoma patients presented different plasma lncRNA levels than healthy donors. Moreover, we found that expression levels of some lncRNAs were linked with better outcomes in patients treated with the BRAF inhibitor. Furthermore, the levels of selected lncRNAs appeared to be linked with primary progression to BRAF inhibitor in melanoma patients.

## RESULTS

### Patients

Between June 2013 and November 2014, 58 patients with *BRAF*-mutated metastatic melanoma began treatment with vemurafenib. All of them had at least one response assessment in addition to available pretreatment plasma collection for further lncRNA analyses. The characteristics of the examined melanoma patients treated with vemurafenib are shown in Table 1.

**Table 1:** Baseline characteristics of BRAF-mutated melanoma patients treated with vemurafenib

Parameters		Cases *n* (%)
Sex	Women	29 (50%)
	Man	29 (50%)
Age (mean)	<54	26 (45%)
	>54	32 (55%)
LDH level	Normal	43 (74%)
	>ULN	11 (19%)
	ND	4 (7%)
Brain metastases	Yes	36 (62%)
	No	22 (38%)
Earlier treatment	Yes	9 (15.5%)
	No	49 (84.5%)
Response to vemurafenib treatment	CR	3 (5%)
	PR	30 (52%)
	SD	17 (29%)
	PD	8 (14%)

CR - Complete Response; PR - Partial Response; SD - Stable Disease; PD - Progressive Disease; ULN – upper laboratory norm; ND – not done.

### Plasma lncRNA expression differ in metastatic melanoma patients and healthy individuals

**Figure 1:**
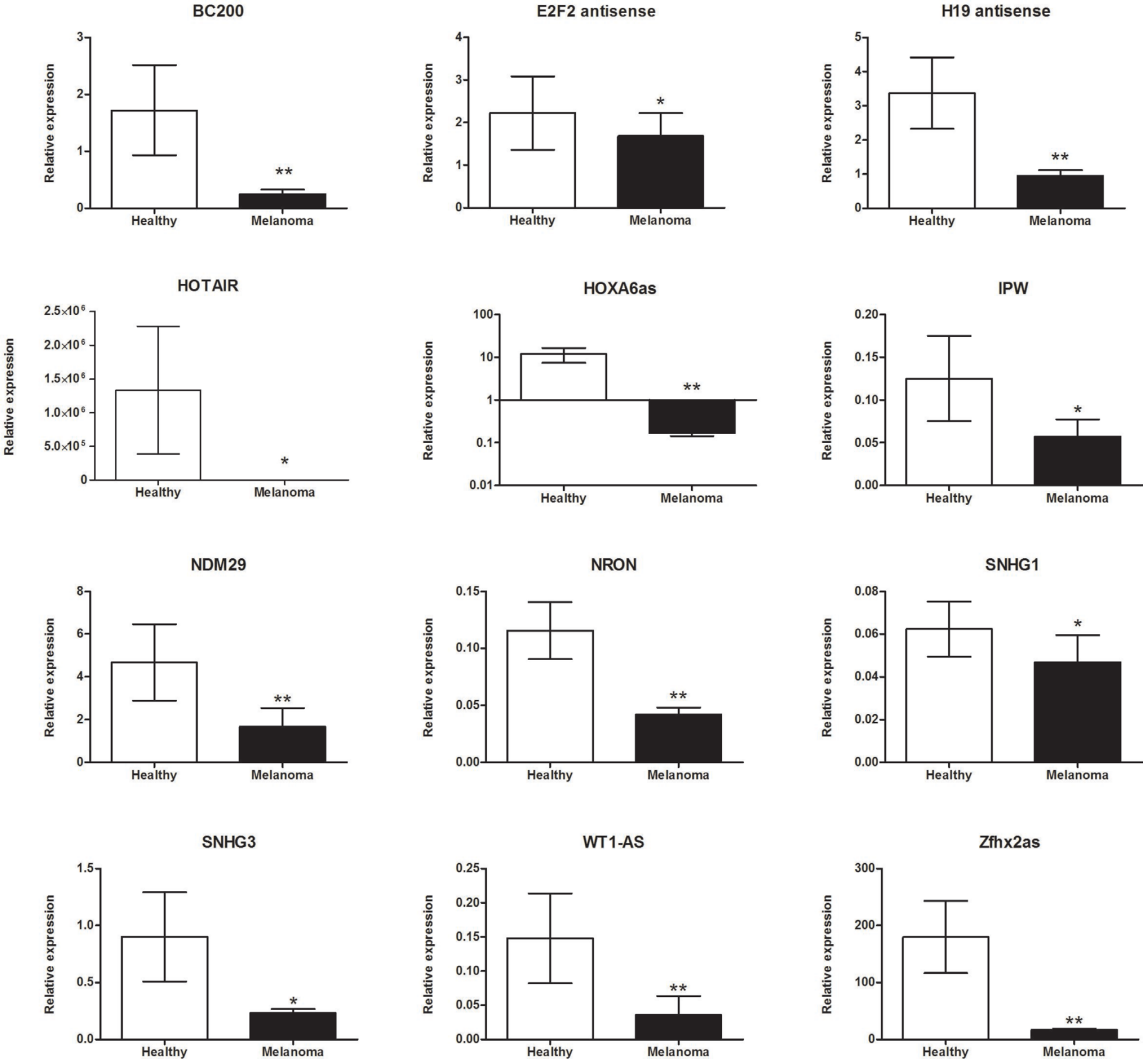
lncRNAs were significantly down-regulated in *BRAF*-mutant metastatic melanoma patients compared to healthy donors. Data present mean expression with standard error (SEM); ^*^p <0.05, ^**^p <0.01.

The expression levels of 90 lncRNAs that were potentially connected with the cancer process was evaluated in 58 BRAF-mutant metastatic melanoma patients and 15 healthy donors (controls) without any histories of cancer or chronic diseases. The plasma from melanoma patients was collected prior to the start of vemurafenib treatment. The expressions of the following 12 lncRNAs were significantly down-regulated in melanoma patients compared to healthy volunteers: (1) brain cytoplasmic (BC)200 (0.251 ± 0.081 versus 1.722 ± 0.792; *p* = 0.004); (2) E2F2 antisense (1.681 ± 0.543 versus 2.223 ± 0.864; *p* = 0.049); (3) H19 antisense (0.956 ± 0.165 versus 3.374 ± 1.047; *p* = 0.001); (4) homeobox transcript (HOTAIR) (8.537 ± 1.026 versus 1.334e+06 ± 9.450e+05; *p* = 0.039); (5) homeobox (HOX)A6as (0.173 ± 0.027 versus 12.02 ± 4.522; *p* = 0.001); (6) imprinted in Prader-Willi Syndrome (IPW) (0.058 ± 0.012 versus 0.125 ± 0.05; *p* = 0.026); (7) neuroblastoma differentiation marker (NDM)29 (1.661 ± 0.882 versus 4.667 ± 1.780; *p* = 0.002); (8) ncRNA repressor of the nuclear factor of activated T-cells (NRON) (0.042 ± 0.006 versus 0.116 ± 0.025; *p* = 0.001); (9) small nucleolar RNA host gene (SNHG)1 (0.025 ± 0.013 versus 0.062 ± 0.013; *p* = 0.029); (10) SNHG3 (0.234 ± 0.034 versus 0.900 ± 0.391; *p* = 0.029); (11) Wilms tumor-antisense (WT1-AS) (0.036 ± 0.027 versus 0.148 ± 0.066; *p* = 0.005); and (12) zinc finger homeobox antisense (ZFHX2AS) (16.51 ± 2.204 versus 179.9 ± 63.48; *p* = 0.002) as shown in Figure 1.

**Figure 2:**
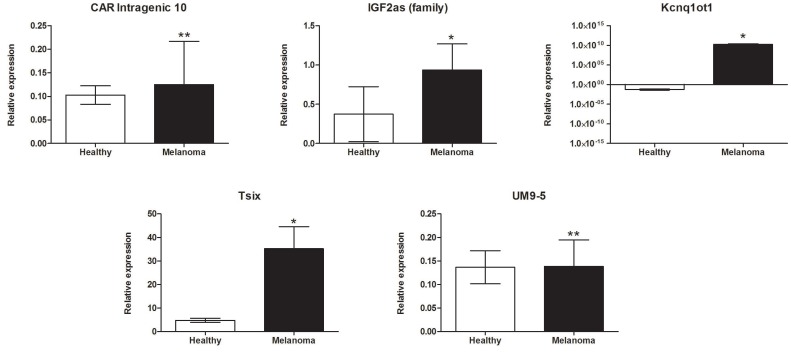
lncRNAs significantly up-regulated in *BRAF*-mutant metastatic melanoma patients compared to healthy donors. Data present mean expression with standard error (SEM); ^*^p < 0.05, ^**^p <0.01.

Expression levels of five lncRNAs were significantly up-regulated in melanoma patients compared to healthy volunteers. Increases in the expressions of several lncRNAs were observed, including chromatin-associated RNA (CAR)-intergenic-10 (0.125 ± 0.091 versus 0.103 ± 0.02; *p* = 0.001), insulin-like growth factor 2-antisense (IGF2as) family (0.935 ± 0.335 versus 0.373 ± 0.351; *p* = 0.025), potassium voltage-gated channel subfamily q overlapping transcript (KCNQ1OT1) (1.755e+010 ± 8.881e+009 versus 0.062 ± 0.023; *p* = 0.028), antisense to X-inactive specific transcript (Tsix) (35.24 ± 9.326 versus 4.795 ± 0.873; *p* = 0.022), and potassium voltage-gated channel subfamily q (UM9-5) (0.139 ± 0.056 versus 0.137 ± 0.035; *p* = 0.002) as shown in Figure 2.

The ROC analysis indicated lncRNA with high sensitivity (from 51.79% to 100%) and specificity (from 50% to 100%) could distinguish cancer from healthy patients. The results of the area under the ROC curve (AUC) analysis were summarized in Table 2.

**Table 2:** lncRNA sensitivity and specificity features for distinguishing cancer and healthy patients; receiver operating characteristic (ROC) curve analysis

lncRNA	AUC	Sensitivity	qSpecificity	*P*-value
CAR Intragenic 10	0.807	68.09	90.91	**<0.0001**
NDM29	0.792	61.54	90.91	**<0.0001**
H19 antisense	0.791	51.79	100.00	**<0.0001**
HOXA6as	0.787	100.00	61.54	**0.0012**
NRON	0.780	76.79	76.92	**0.0001**
Zfhx2as	0.769	100.00	50.00	**0.0004**
IPW	0.767	53.66	100.00	**0.0008**
BC200	0.763	64.15	84.62	**0.0002**
UM9-5	0.759	72.73	80.00	**<0.0001**
WT1-AS	0.753	95.45	61.54	**0.0038**
Kcnq1ot1	0.735	72.73	77.78	**0.0027**
Tsix	0.731	56.25	100.00	**0.0003**
IGF2as (family)	0.707	51.79	91.67	**0.0141**
SNHG3	0.703	83.64	50.00	**0.0219**
SNHG1	0.696	76.79	61.54	**0.0164**
E2F2 antisense	0.692	65.31	72.73	**0.0186**
HOTAIR	0.681	58.14	80.00	**0.0221**

### Correlation of plasma lncRNA expression with response to vemurafenib treatment in BRAF-mutant metastatic melanoma patients

**Figure 3:**
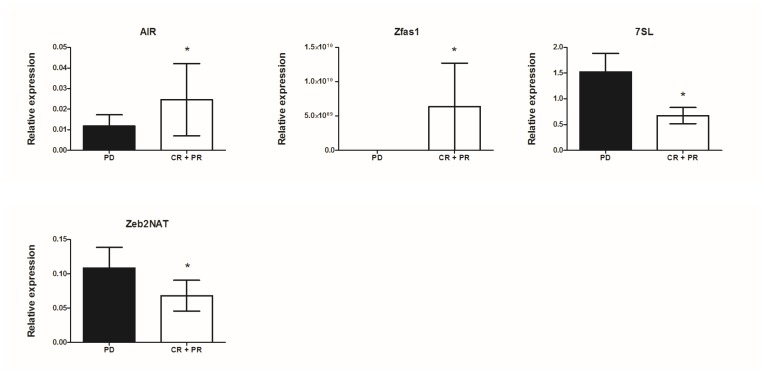
Correlation of lncRNA expression with response to vemurafenib treatment in metastatic melanoma patients; mean expression with standard error (SEM); ^*^p < 0.05, ^**^p <0.01.

Associations between the expression level of lncRNAs and patients’ response to vemurafenib treatment were analyzed. Patients were grouped according to treatment response (Table 1). The lncRNA expression levels in patients developing progressive disease (PD) were compared to those with objective response rates (complete and partial response [CR+PR] groups). Significant differences between the PD and CR+PR groups were observed for antisense of IGF2R non-protein coding RNA (AIR) (0.012 ± 0.005 versus 0.025 ± 0.018; *p* = 0.038), antisense to zinc finger NFX1 (Zfas1) (0.164 ± 0.038 versus 0.649 ± 0.24; *p* = 0.022), 7SL (1.522 ± 0.363 versus 0.673 ± 0.157; *p* = 0.030), and zinc finger AE-binding homeobox 2-natural antisense transcript (Zeb2NAT) (0.109 ± 0.03 versus 0.068 ± 0.022; *p* = 0.045) as shown in Figure 3.

In the subgroups (PD versus CR+PR) presenting significant differences in lncRNA expression level the discrimination ratio was assessed using the ROC analysis, which showed AIR (AUC = 0.842; p<0.0001), Zfas1 (AUC = 0.766; *p* = 0.001), 7SL (AUC = 0.787; *p* = 0.006), and Zeb2NAT (AUC = 0.747; *p* = 0.008) can differentiate between CR+PR and PD groups.

### Plasma lncRNA expression level as biomarker of progression-free survival and overall survival in BRAF-mutated metastatic melanoma patients treated with vemurafenib

**Figure 4:**
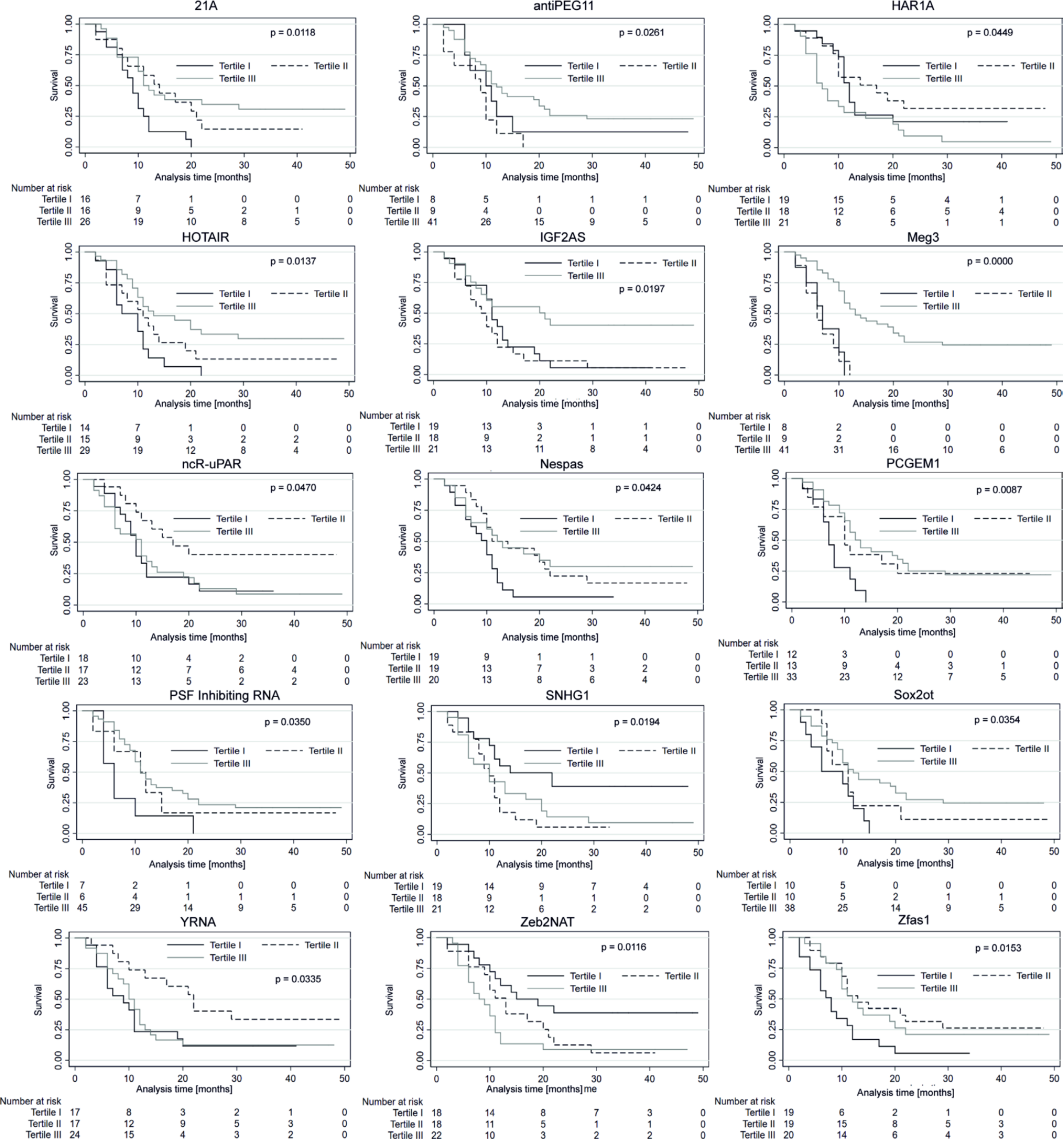
Progression-free survival probability curves of the three subgroups stratified based on low, medium and high expression of lncRNAs. Kaplan–Meier survival estimates test; *p* < 0.05 considered as significant.

Each of the 58 patients included in the analysis was classified into one of three sub-groups depending on the relative plasma lncRNA level (low, medium, or high). The groups were stratified by dividing the observed samples into tertiles as shown in Table 3. The median follow-up duration was 11 months. The median PFS and OS in all studied patients were eight and 11 months, respectively. The expression levels of lncRNA in particular groups (Tertile I–III) was correlated with PFS and OS. In patients with high expression (Tertile III) of antiPEG11 (*p* = 0.018), HOTAIR (*p* = 0.034), IGF2AS (*p* = 0.015), maternally expressed gene (MEG)3 (*p* = 0.0004), prostate-specific transcript (PCGEM1 (*p* = 0.027), and polypyrimidine tract-binding protein-associated splicing factor (PSF) inhibiting RNA (*p* = 0.039), significantly longer PFS was observed compared to patients with low expression (Tertile I) of the corresponding lncRNAs. In patients with medium (Tertile II) level of lncRNAs, 21A (*p* = 0.021), lincRNA-RoR (*p* = 0.032), Y-RNA (*p* = 0.005), and Zfas1 (*p* = 0.028), a significantly longer patient PFS was observed compared to patients with high expression (Tertile III) of the corresponding lncRNAs. Patients with low (Tertile I) levels of Zeb2NAT showed longer PFS compared to patients with high (Tertile III) Zeb2NAT levels (*p* = 0.0004, Figure 4).

**Table 3:** The plasma lncRNA levels in melanoma patients displaying low, medium, and high expressions

lncRNA	Low (tertile I)	Medium (tertile II)	High (tertile III)
antiPEG11	1.11E-16 - 1.43E-04	1.43E-04 - 2.51E-03	2.51E-03 - 2.35E+11
HOTAIR	1.50E-11 - 5.25E+00	5.25E+00 - 8.66E+00	8.66E+00 - 3.38E+01
IGF2AS	9.39E-16 - 2.27E-02	2.27E-02 - 3.46E-01	3.46E-01 - 1.53E+01
Meg3	2.78E-17 - 1.32E-03	1.32E-03 - 1.08E-02	1.08E-02 - 2.08E+11
PCGEM1	2.62E-06 - 2.74E-03	2.74E-03 - 3.63E-02	3.63E-02 - 2.35E+11
PSF inhibiting RNA	2.78E-14 - 2.21E-01	2.21E-01 - 1.58E+11	1.58E+11 - 2.39E+11
21A	2.74E-12 - 7.38E-01	7.38E-01 - 2.92E+00	2.92E+00 - 1.06E+02
lincRNA-RoR	1.20E-15 - 3.23E+00	3.23E+00 - 3.30E+02	3.30E+02 - 2.08E+11
Y-RNA	1.70E-08 - 1.34E+02	1.34E+02 - 3.83E+02	3.83E+02 - 2.23E+03
Zfas1	1.16E-13 - 1.56E-01	1.56E-01 - 3.93E-01	3.93E-01 - 2.04E+11
Zeb2NAT	3.15E-13 - 2.56E-02	2.56E-02 - 8.19E-02	8.19E-02 - 2.08E+11
Nespas	2.39E-13 - 6.67E-02	6.67E-02 - 1.39E-01	1.39E-01 - 2.04E+11
Sox2ot	4.18E-13 - 6.29E-03	6.29E-03 - 3.88E-02	3.88E-02 - 2.35E+11
HAR1A	2.98E-14 - 8.23E-03	8.23E-03 - 3.81E-02	3.81E-02 - 1.91E-01
ncR-uPAR	6.70E-13 - 3.73E-02	3.73E-02 - 2.09E-01	2.09E-01 - 5.07E+00
SNHG1	2.74E-13 - 8.19E-03	8.19E-03 - 3.97E-02	3.97E-02 - 6.66E-01

**Figure 5:**
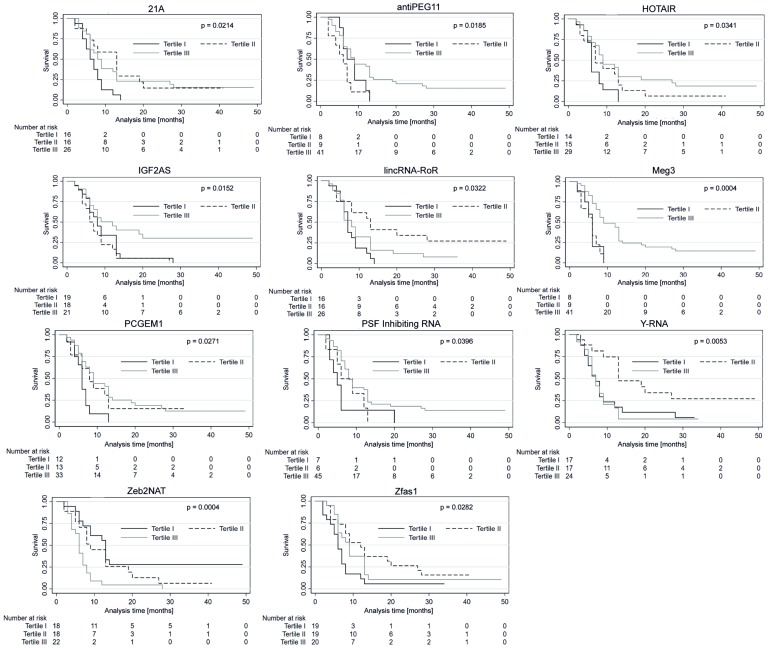
Overall survival (OS) probability curves of the three subgroups stratified based on low, medium and high expression of lncRNAs. Kaplan-Meier survival estimates test; *p* < 0.05 considered as significant.

The OS of patients with high levels (Tertile III) of antiPEG11 (*p* = 0.026), HOTAIR (*p* = 0.013), IGF2AS (*p* = 0.019), maternally expressed gene (MEG)3 (*p* = 0.0000), neuroendocrine secretory protein antisense (Nespas) (*p* = 0.042), PCGEM1 (*p* = 0.008), PSF inhibiting RNA (*p* = 0.035) and sex determining region-overlapping transcript (SOX2ot) (*p* = 0.035) was significantly longer than in patients with low (Tertile I) level of corresponding lncRNAs. With respect to lncRNAs, 21A (*p* = 0.011), HAR1A (*p* = 0.044), ncR-uPAR (*p* = 0.047), Y-RNA (*p* = 0.033), and Zfas1 (*p* = 0.015), a significantly longer OS was observed in patients with medium compared to high levels of corresponding lncRNAs (Tertile II versus III). In patients with low (Tertile I) small nucleolar RNA host gene (SNHG1) (*p* = 0.019) and Zeb2NAT (*p* = 0.011) expressions, longer OS was seen compared to patients with high levels (Tertile III) of corresponding lncRNAs (Figure 5).

We next evaluated the prognostic value of the differentially expressed lncRNAs using univariate and multivariate Cox regression analyses. The univariate analysis showed that lactate dehydrogenase (LDH) serum levels, brain metastases, earlier systemic treatment, and lncRNA serum levels of antiPeg11, IGF2AS, MEG3, SOX2ot, and Zeb2NAT were significantly associated with patient PFS and OS as shown in Table 4. The above-mentioned parameters were further analyzed using a multivariate analysis, and antiPeg11, IGF2AS, MEG3, SOX2ot, and Zeb2NAT were identified as statistically significant prognostic factors (Table 5).

**Table 4:** Univariate Cox regression analysis in *BRAF*-mutant melanoma patients treated with vemurafenib

Parameters	Categories	Progression-free survival			Overall survival		
		*P*-value	HR	95%CI	*P*-value	HR	95%CI
Age	>54 vs <54	0.0582	1.03	1.00–1.07	0.0526	1.04	1.00–1.07
LDH	>ULN vs normal	**0.0431**	3.11	1.12– 5.93	**0.0100**	3.23	1.32–7.89
Brain metastases	yes vs no	**0.0273**	2.48	1.11–5.55	**0.0031**	3.81	1.57–9.22
Earlier treatment	yes vs no	**0.0387**	3.23	1.06–9.85	**0.0144**	3.89	1.31–11.56
Gender	male vs female	0.7799	1.11	0.54–2.27	0.4494	1.33	0.63–2.81
antiPeg11 expression	high vs low	**0.0486**	3.11	1.65–8.86	**0.0460**	3.31	1.64–9.16
antiPeg11 expression	high vs medium	**0.0414**	3.22	1.75–8.69	**0.0481**	3.83	1.65–10.14
HOTAIR expression	high vs low	0.3843	0.54	0.13–2.17	0.9114	1.07	0.32–3.62
HOTAIR expression	high vs medium	0.7965	0.88	0.34–2.28	0.8072	0.88	0.32–2.41
IGF2AS expression	high vs low	**0.0460**	3.37	1.93–8.90	**0.0490**	3.52	1.90–11.06
IGF2AS expression	high vs medium	**0.0301**	4.04	1.89–6.31	**0.0324**	4.66	1.91–12.75
MEG3 expression	high vs low	**0.0499**	3.10	2.01–7.09	**0.0393**	3.02	1.63–10.73
MEG3 expression	high vs medium	**0.0426**	3.41	1.04–11.19	**0.0107**	2.52	1.45–17.34
Nespas expression	high vs low	0.7308	0.83	0.29–2.41	0.4067	1.55	0.55–4.39
Nespas expression	high vs medium	0.6017	0.80	0.35–1.85	0.7108	0.85	0.36–1.99
PCGEM1 expression	high vs low	0.6222	1.36	0.40–4.56	0.3339	1.85	0.53–6.4
PCGEM1 expression	high vs medium	0.6999	0.82	0.29–2.30	0.3569	1.59	0.59–4.26
PSFinhibitingRNA expression	high vs low	0.3375	1.83	0.53–6.29	0.2861	1.92	0.58–6.38
PSFinhibitingRNA expression	high vs medium	0.7645	0.77	0.14–4.29	0.0578	0.20	0.04–1.05
Sox2ot expression	high vs low	**0.0450**	3.86	1.64–10.75	**0.0190**	4.15	1.93–12.78
Sox2ot expression	high vs medium	0.4880	3.17	0.91–9.74	**0.0147**	3.05	1.95–11.05
SNHG1 expression	high vs low	0.1722	0.48	0.17–1.38	**0.0314**	0.42	0.19–0.92
SNHG1 expression	high vs medium	0.8663	1.09	0.40–2.96	0.3473	1.42	0.68–2.95
Zeb2NAT expression	high vs low	**0.0039**	0.24	0.09–0.63	**0.0015**	0.29	0.13–0.62
Zeb2NAT expression	high vs medium	**0.0268**	0.33	0.12–0.88	**0.0491**	0.34	0.26–0.72

HR – hazard ratio; CI – confidence interval; LDH – lactate dehydrogenase; ULN – upper laboratory norm

**Table 5:** Multivariate Cox regression analysis in *BRAF*-mutant melanoma patients treated with vemurafenib

Parameters	Categories	*P*-value	HR	95%CI	*P*-value	HR	95%CI
antiPeg11 expression	high vs low	0.5702	1.43	0.41–4.98	0.4458	0.54	0.11–2.62
antiPeg11 expression	high vs medium	**0.0008**	7.14	2.26–22.57	**0.0131**	4.97	1.40–17.66
MEG3 expression	high vs low	**0.0071**	4.67	3.82–12.75	**0.0270**	4.21	1.18–15.04
MEG3 expression	high vs medium	**0.0001**	14.09	3.78–52.51	**<0.0001**	121.09	17.64–831.43
Sox2ot expression	high vs low	**0.0008**	9.49	2.54–35.39	**0.0001**	24.44	5.11–116.97
Sox2ot expression	high vs medium	0.1035	2.41	0.84–6.92	**0.0056**	6.49	1.73–24.40
Zeb2NAT expression	high vs low	**0.0209**	0.34	0.03–0.71	**0.0025**	0.32	0.17–0.68
Zeb2NAT expression	high vs medium	**0.0168**	0.31	0.13–0.81	**0.0441**	0.30	0.24–0.74
IGF2AS expression	high vs low				**0.0480**	3.89	1.99–16.31
IGF2AS expression	high vs medium				**0.0118**	5.34	1.45–19.69

HR: hazard ratio; CI: confidence interval.

Table 6 lists the correlation between antiPeg11, IGF2AS, MEG3, SOX2ot, and Zeb2NAT expression levels in serum and the clinical characteristics of *BRAF*-mutated advanced melanoma patients treated with vemurafenib. A higher level of plasma Zeb2NAT correlated with the occurrence of brain metastases (*p* = 0.032). Moreover, a higher level of plasma SOX2ot correlated with male gender (*p* = 0.030). However, we did not observe any association between the expressions of the remaining lncRNA and patients ages, sexes, LDH levels, occurrence of brain metastases, and/or earlier applied systemic treatment.

**Table 6:** Correlation between plasma lncRNA concentrations and clinical characteristics of *BRAF*-mutant advanced melanoma patients treated with vemurafenib (median [interquartile range])

Parameters	Totalcases	antiPeg11	*P*-value	IGF2AS	*P*-value	MEG3	*P*-value	Sox2ot	*P*-value	Zeb2NAT	*P*-value
		95%CI		95%CI		95%CI		95%CI		95%CI	
**Age(years)**											
<54	26	0.0007412–0.005661	0.1700	0.01906–3.932	0.7600	–5.79E+09–1.43E+10	0.6700	0.005765–0.04132	0.9700	–0.1744–0.8754	0.2900
>54	32	–4.03E+10–9.90E+10		0.2292–0.9445		–2.34E+10–5.93E+10		–2.97E+10–7.68E+10		0.03009–0.08331	
**Sex**											
Female	29	–1.70+E10–7.19E+10	0.8931	0.1518–1.756	0.4589	8.70E+10–1.03E+09	0.1480	0.005459–0.02705	**0.0308**	–5.93E+09–2.83E+10	0.8773
Male	29	–1.95E+10–5.32E+10		–0.2154–2.046		–1.67E+10–1.03E+11		–1.48E+10–7.75E+10		–0.1224–0.6419	
**Serum LDH**											
Normal	43	–1.11E+10–6.03E+10	0.7211	0.3018–2.122	0.7404	1.76E+10–1.04E+11	0.0773	–8.76E+09–4.87E+10	0.4687	–5.31E+09–1.57E+10	0.1375
>ULN	11	–2.47E+10–5.61E+10		0.03346–0.389		–0.001403–0.00511		–0.003591–0.04122		–1.19E+10–3.08E+10	
Brain metastases											
Yes	36	–1.05E+10–2.77E+10	0.3173	–0.2635–1.982	0.1224	–1.31E+10–8.01E+10	0.3516	0.003503–0.02831	0.0523	–8.30E+09–3.85E+10	**0.0329**
No	22	–1.49E+10–7.82E+10		0.09858–1.854		2.02E+09–1.01E+11		–1.26E+10–6.75E+10		0.03328–0.07773	
**Earlier treatment**											
Yes	9	–5.88E+09–5.96E+10	0.9215	0.2937–1.885	0.3104	8.62E+09–8.43E+10	>0.9999	–7.28E+09–4.10E+10	0.3587	–3.27E+09–1.61E+10	0.3226
No	49	–0.001655–0.005927		–0.01375–0.2662		–5.74E+10–1.22E+11		–0.02217–0.05198		0.02444–0.1843	

LDH – lactate dehydrogenase; ULN – upper laboratory norm; CI – confidence interval.

## DISCUSSION

This study included three main findings: (1) plasma lncRNA levels differed in BRAF-mutated advanced melanoma patients and healthy controls; (2) expression levels of selected lncRNA were linked with favorable median PFS and OS of patients treated with BRAF inhibitor; and (3) plasma levels of particular lncRNAs were linked with primary progression of the disease in patients treated with BRAF inhibitor.

Although some driver mutations in *BRAF* or *NRAS* genes have been identified in melanoma, the efficiencies of their inhibitors are still limited [18–22]. The BRAF mutations occur in about 50% of patients with skin melanoma. These patients can be treated with BRAF inhibitors (vemurafenib or dabrafenib) alone or in combination with MEK inhibitors (cobimetinib or trametinib), which is the currently recommended treatment plan. The objective response rate (CR and PR) is observed in 50% to 70% of patients; however, about half of them will develop resistance to the treatment after 6 to 11 months of therapy [3, 5, 21, 23].

Cancer diagnosis and prognosis employing circulating lncRNAs are preferable when compared to classical biopsies of tumor tissues, especially due to their noninvasiveness and their increasing potential for routine use in clinical practice. LncRNAs can be actively released by tumor tissues and cells [27]. However, elevated quantities of lncRNAs in plasma may originate from multiple sources, including cancer-adjacent normal cells, immune cells, and other blood cells [28, 29]. In our study we identified 12 downregulated and five upregulated plasma lncRNAs in BRAF-mutated metastatic melanoma compared to healthy individuals. The ROC analysis indicated lncRNA with high sensitivity and specificity for distinguishing between cancer and healthy patients. To our knowledge, this is the largest study showing the difference in the expression profile of circulating lncRNAs in *BRAF*-mutated melanoma patients and healthy donors. These data show that dysregulated plasma lncRNAs can help make diagnoses of *BRAF*-mutated advanced melanoma with high sensitivity and specificity. However, evaluation of the above specified dysregulated plasma lncRNAs in the primary diagnosis of melanoma needs further investigation. In addition, circulating IGF2as was identified as an independent factor for *BRAF*-mutated advanced melanoma prognosis in patients receiving vemurafenib.

Elevated serum LDH, presence of brain metastases, and earlier systemic treatment are well known negative prognostic markers in melanoma regardless of the applied treatment [11, 21, 22]. In our study, these factors were linked with poor OS in the univariate analysis. However, they lost their significance in the multivariate analysis probably due to the small sample size. The Kaplan-Meier curves showed that higher plasma levels of antiPeg11 (PFS: 9 versus six months; OS: 12 versus 9 months), IGF2AS (PFS: 12 versus six months; OS: 21 versus nine months), and MEG3 (PFS: 9 versus 6 months; OS: 13 versus 6 months) were linked with longer median PFSs and OSs compared to lower levels. The higher SOX2ot plasma level was linked to longer median OS (12 versus 6 months) but not median PFS. Moreover, low levels of Zeb2NAT were linked with longer median PFS (13 versus 6 months) and median OS (15 versus 8 months) compared to higher levels. Furthermore, univariate and multivariate Cox regression models showed that elevated baseline plasma levels of antiPeg11, MEG3, and SOX2ot and decreased level of Zeb2NAT appear to be positive prognostic factors linked with longer PFS and OS. However, higher levels of plasma SOX2ot might be also linked with female gender. The univariate analysis also showed that higher expression level of IGF2as was linked with longer PFS and OS, however the multivariate analysis showed significant difference only in OS. When taken together, these results demonstrate that lncRNA IGF2AS, antiPeg11, MEG3, and Zeb2NAT appear to be independent prognostic factors in *BRAF*-mutated advanced melanoma patients treated with vemurafenib.

The function of theses circulating lncRNAs is still unknown. Moreover, they probably are derived from tumor cells; however, their origin from inflammatory cells is also possible. Zeb2NAT lncRNA is a regulator of Zeb2, one of the major transcription factors involved in epithelial-mesenchymal transition (EMT) and was shown to directly represses E-cadherin during epithelial–mesenchymal transition (EMT) [24]. There are two mechanisms involved in the regulation of Zeb2 expression: (1) at the transcriptional level and (2) at the posttranscriptional level by lncRNA Zeb2NAT. It was shown that cancer-associated fibroblasts secrete transforming growth factor (TGF)-beta1 that up-regulated Zeb2NAT, leading to Zeb2 activation and EMT induction that was responsible for bladder cancer cell invasion [24]. It was also shown that Zeb2 is involved in acquired resistance to the BRAF inhibitor in *BRAF*-mutated melanoma. Mechanistically, it was shown that the BRAF inhibitor induces activation of Zeb2, which stimulates Mer tyrosine kinase (MerTK) through target of rapamycin complex (TORC)1-triggered activation of autophagy leading to secondary resistance to BRAF inhibition and melanoma cell growth [25]. It was previously shown that IGF2 promotes cancer development and progression [26]. Upregulation of IGF2anti-sense inhibits IGF2 in murine neurons [27, 28] and human non-small cell lung cancer (NCSLC) cell lines [29]. In NCSLC, upregulation of IGF2as inhibited vascular endothelial growth factor (VEGF) and basic fibroblast growth factor (bFGF) expressions probably through IGF2 inhibition [29]. It was shown that in NSCLC patient tissues, downregulated IGF2as expression was linked with much worse OS than in NSCLC patients with upregulated IGF2as expression [29]. In our study, IGF2as was upregulated in BRAF-mutated advanced melanoma patients compared to healthy donors, and its high expression level was linked to more favorable survival. However, the function of circulating IGF2as in cancer is still unknown. Upregulation of MEG3 inhibited melanoma cell proliferation, invasion, and migration, enhanced melanoma cell apoptosis, and arrested melanoma cell cycle. Overexpression of MEG3 suppressed the growth of xenograft tumors and improved chemotherapy sensitivity of A375 cells to cisplatin and 5-FU treatment [30]. However, there is a lack of data concerning circulating MEG3 in cancer. In our study, high levels of plasma MEG3 were linked with longer survival compared to low levels of this lncRNA. The role and source of circulating MEG3 in cancer patients is unknown. To our knowledge, this is the first report on circulating MEG3 in cancer patients. Another dysregulated lncRNA in our analysis was antiPeg11. To our knowledge, antiPeg11 has not yet been described, so its function in cancer is unknown. SOX2ot is upregulated and appears to function a an oncogene in multiple types of cancers; however, some studies show that SOX2ot may play a tumor suppressor role [31–37]. It was shown that low serum expressions of SOX2ot were associated with longer OS. However, this study was performed in NCSLC patients in the Chinese population [38]. To our knowledge, SOX2ot has not been previously described in melanoma patients. These reports further strengthen our finding that circulating lncRNA IGF2AS, antiPeg11, MEG3, and Zeb2NAT have immense potential to serve as biomarkers in patients treated with BRAF inhibitors.

Primary progression at first tumor assessment, performed after two months of vemurafenib therapy, was linked with pretreatment plasma upregulation of 7SL and Zeb2NAT and downregulation of Zfas1 and AIR. Inversely, downregulation of 7SL and Zeb2NAT, and upregulation of Zfas1 and AIR was linked with objective responses (CR+PR) to therapy. Most of the patients responded to vemurafenib treatment. In our study, CR and PR were noted in 56% of patients, while stable disease was observed in 29% and primary progression in 14% of patients. Zfas1 is an lncRNA that has recently been reported to function as a potential oncogene by promoting cell proliferation and metastasis in several human cancers [39–42]. High Zfas1 expression has been proven as an unfavorable prognostic biomarker for many types of cancers; however, in melanoma it has not yet been described [41, 43–48]. Furthermore, in breast cancer Zfas1 inhibited cell proliferation, migration, invasion, and the EMT process. It was shown that Zfas1 overexpression inhibited cell proliferation by arresting the cell cycle at the G0/G1 phase and promoting cell apoptosis. In breast cancer cells overexpressing Zfas1, the EMT-related markers, such as E-cadherin expression, were upregulated while N-cadherin and vimentin expressions were downregulated, indicating that the effects of Zfas1on cell migration and invasion were partially associated with the EMT process [49]. Another upregulated lncRNA-linked primary progression was 7SL. This lncRNA was found to be over-expressed in many tumors [50] and promoted growth of cancer cells by repressing P53 translation [51]. However, to our knowledge it has not been described in melanoma. Also the downregulated AIR was not yet characterized. These four lncRNAs (Zeb2NAT, Zfas1, 7SL, and AIR) might be involved in mechanisms of resistance to vemurafenib and serve as biomarkers of therapy ineffectiveness. However, further studies in a larger cohort are needed to confirm these findings. The function of the above lncRNA in patients who are non-responding to vemurafenib needs further evaluation.

## CONCLUSIONS

Evaluation of plasma lncRNA expression levels for advanced melanoma diagnosis and prognosis evaluation is a safe and valuable method; however, it needs further validation in larger cohorts and randomized trials. We show that lncRNA IGF2AS, MEG3, and Zeb2NAT are independent prognostic factors in *BRAF*-mutated advanced melanoma patients treated with vemurafenib. Further validation of these biomarkers in larger cohorts of patients receiving a BRAF inhibitor combined with a MEK inhibitor is needed. The next step could include the evaluation of these circulating lncRNAs as predictive factors in a randomized study including melanoma patients receiving BRAF+MEK inhibitors and immunotherapy.

## MATERIALS AND METHODS

### Patients

This retrospective study included BRAF mutant metastatic melanoma patients treated at one Polish oncology center. All patients received a BRAF inhibitor (vemurafenib) in a dose of 960 mg twice daily. Blood samples were drawn from 58 melanoma patients and 15 healthy donors without histories of cancer or chronic diseases.

All donors were informed about the aim of the study, use of personal data, and genetic data protection. They agreed to use their material and they filled the consent form. The study methods conformed to the standards set by the Declaration of Helsinki and did not violate the rights of other persons or institutions. The bioethical committee approved the experimental study (agreement No. 13/2008).

Whole blood was collected into tubes containing ethylenediaminetetraacetic acid (EDTA) (SARSTED Monovette EDTA K) and immediately centrifuged (10 min at 1900 × g, room temperature [RT]). The upper plasma phase was transferred to a new tube without disturbing the intermediate buffy coat layer. Next, the plasma samples were centrifuged (10 min at 16,000 x g, RT) to remove cellular nucleic acids attached to cell debris, transferred to new tubes, and stored at –80°C until use.

Overall survival (OS) was calculated from the date of vemurafenib treatment initiation to the date of death from any cause. Patients who were still alive were censored at the last follow-up. Progression-free survival (PFS) was calculated from the date of initiation of vermurafenib therapy until progression as documented by imaging according to response evaluation criteria in solid tumors (RECIST), clinical examination, or death. Those who were alive and without progression were censored at the last follow-up.

### RNA isolation

Total RNA was isolated from plasma samples using miRNeasy Serum/Plasma Kit (Qiagen) according to the isolation protocol for total RNA. The quality and quantity of RNA samples were checked with a NanoDrop spectrophotometer (Thermo Scientific), and samples were stored at –80° C until use.

### cDNA synthesis and qRT-PCR reaction

In this study, the 90 lncRNAs, potentially connected with cancer and well-annotated and registered in the lncRNA database (www.lncrnadb.org), were analyzed using the commercially available LncProfiler qPCR Array Kit (SBI).

Reverse transcription was performed according to the manufacturer’s protocol and was based on three steps: i) poly-A tailing; ii) annealing anchor dT adaptor; and iii) cDNA synthesis.

cDNA was used for the qRT-PCR reaction using LightCycler 480 SYBR Green I Master buffer (Roche) and lncRNA primers from Primer Plate (component of the LncProfiler qPCR Array Kit) according to the manufacturer’s protocol by the LightCycler 96 (Roche). All qRT-PCR data were analyzed by calculating the ΔC_t_, normalized against mean expression of anti-nitric oxide synthase (NOS)2A+human accelerated region (HAR)1B+taurine upregulated gene (TUG)1, which were the most stable transcripts in all of the examined samples (healthy and cancer) with the lowest C_t_s variation compared to the reference genes from the LncProfiler qPCR Array Kit (SBI). The fold-change of lncRNA expression was determined by equation 2^–ΔCt^ and compared to the appropriate group.

### Statistical analysis

Statistical analysis was performed with MedCalc version 10.3.2. (MedCalc Software, Mariakerke, Belgium) and Statistica 12 (StatSoft Inc., Poland). All data are presented as means + standard error (SEM). The lncRNAs expression profiles were compared between melanoma patients and controls.

Furthermore, the melanoma patients were grouped into similar clinical categories. The comparison of the lncRNAs expression profiles between analyzed groups was done by Student’s *t*-test or the Mann-Whitney test if data did not follow a normal distribution. Normality was analyzed with the Shapiro-Wilks test. Receiver operating characteristics (ROC) curves were calculated. An optimal cut-off point was calculated according to the highest accuracy (minimal false negative and false positive rates). The area under the ROC curve (AUC) was used to assess the prognostic properties of each lncRNA. Kaplan-Meier survival curves for the analyzed groups were plotted, and the log-rank test was used to compare the survival probability curves. For further analyses, the samples ordered based on their lncRNA levels were stratified into three groups of lncRNA expression: (1) low; (2) medium; and (3) high based on the three tertiles as shown in Table 3. A Cox proportional hazards regression model was used to determine which lncRNAs were significantly associated with both OS and PFS. The results were expressed as the hazard ratios (HRs) and 95% confidence intervals (CIs). All of the tests were performed as two-tailed tests and were considered significant at *p* < 0.05.

### Ethics approval

Study is based on human blood samples. All donors were informed about the aim of the study, personal data and genetic data protection, and they agreed to use their material. The experiments were undertaken with the understanding and written consent of each subject. The study methodologies conformed to the standards set by the Declaration of Helsinki. Study does not violate the rights of other persons or institutions. Bioethical committee approved this study - agreement No. 13/2008.
